# Bipolar disorder-associated variants in RAB5A disrupt its function in endolysosomal trafficking in fruit fly and mammalian systems

**DOI:** 10.21203/rs.3.rs-10538807/v1

**Published:** 2026-08-24

**Authors:** Jimmy Holder, Jonathan Andrews, Basabdatta Adhikari, Michael Wangler, Jill Rosenfeld, Rebekah Townsley, Saurabh Srivastav

**Affiliations:** Baylor College of Medicine; Baylor College of Medicine; Baylor College of Medicine; Baylor College of Medicine; Baylor College of Medicine

## Abstract

Bipolar disorder is a severe neuropsychiatric disorder associated with significant morbidity and mortality. Although the heritability of bipolar disorder is among the highest of all neuropsychiatric disorders, the specific genetic etiologies for most affected individuals have remained elusive. This is due, in part, to difficulty confirming pathogenicity of genomic variants identified in bipolar disorder populations. From the Bipolar Exomes Browser (BipEX) project, we identified three missense variants in *RAB5A* in four individuals with bipolar disorder not found in controls. We used a *Drosophila* overexpression system to evaluate these bipolar disorder-associated variants in *RAB5A*. We determined that when bipolar disorder-associated *RAB5A* variants are expressed in fruit flies, synaptic neurotransmission, endolysosomal trafficking, viability, survival and wing morphology are all disrupted. We confirmed that certain bipolar disorder-associated *RAB5A* variants also disrupt early endosomal trafficking in mammalian cells. This confirms bipolar disorder-associated variants in *RAB5A* disrupt its normal function. Moreover, *Drosophila melanogaster* is a viable experimental system to test the pathogenicity of genomic variants associated with psychiatric disorders.

## Introduction

Bipolar disorder has one of the most robust inheritances of the neuropsychiatric disorders with twin studies suggesting a heritability as high as 87%^1^. Despite this, the specific genomic variants driving bipolar disorder have been elusive. However, significant progress has recently been made in cataloguing genomic variants in populations of individuals with bipolar disorder through next generation sequencing. One of the largest gene discovery efforts sequenced over 14,000 affected individuals and controls^2^. While this study identified many new candidate genes for bipolar disorder, whether these genomic variants alter the function of proteins encoded by their respective genes and lead to neuronal dysfunction are largely unknown.

We previously reported a genomic variant identified from multiple individuals with bipolar disorder, *SHANK3* duplication, and confirmed that a mouse model of this disorder results in an array of behavioral abnormalities that mimic manic-like behavior^3^. We then investigated the consequences of this variant on neuronal dysfunction, electrophysiologic abnormalities and the molecular pathways with which SHANK3 interacts. From this starting point, we compared those proteins with which SHANK3 interacts with candidate bipolar disorder genomic variants. We identified *RAB5A* as a gene whose protein product interacts with SHANK3^3^ and in which there are multiple missense variants in individuals with bipolar disorder^2^.

*Drosophila* models have been previously generated to explore the mechanisms underlying bipolar disorder, focusing on endophenotypes such as hyperactivity, sleep disruption, and increased sensitivity to rewarding stimuli^4,5^. One of the earliest models investigated the glycogen synthase kinase-3 (GSK-3) ortholog *shaggy*, which interacts with lithium^6^ and is a significant contributor to the generation of a circadian rhythmicity^7^ and aggressive patterns of behavior in flies^8^. As more genes have been identified which putatively contribute to bipolar disorder, multiple candidate genes linked to neurotransmitter pathways and receptors have emerged from case-control and family-based studies^9^.

This makes the small GTPase Rab5 of particular interest, as it has been shown to affect several critical processes key to neurotransmission and neuronal function in *Drosophila*. While ubiquitously expressed, *Rab5* is enriched in the larval and adult brain^10^. It is a significant contributor to neurotransmitter release and synaptic vesicle recycling, making it important for synaptic activity^11–13^. *Rab5* has also been implicated in a number of other neuronal processes, including axon elongation and dendrite branching^14,15^, synaptic vesicle size^11,16^ and neuronal survival^17,18^. *Drosophila* models have also confirmed *Rab5* is a significant contributor to the pathogenesis of ALS and Huntington disease^19,20^, but little is known about how *Rab5* contributes to other neuropsychiatric diseases. To determine if *RAB5A* bipolar disorder-associated variants alter its protein function, we expressed reference and variant sequences of *RAB5A* in *Drosophila melanogaster* and screened for changes in neuronal electrophysiology, sub-cellular organization, adult viability, survival, body morphology and behavior.

## Results

### Identification and bioinformatic characterization of bipolar disorder-associated RAB5A variants

**Figure 1:**
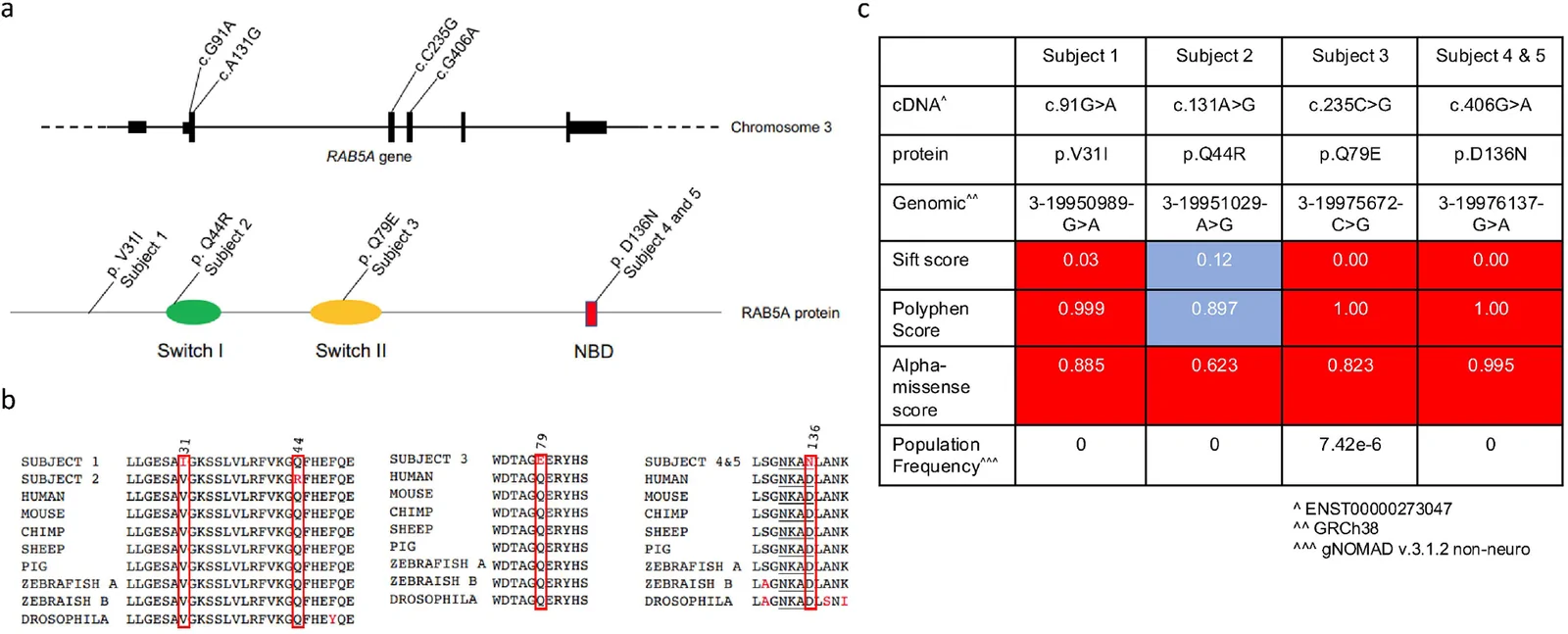
*RAB5A* variants associated with bipolar disorder. a. Diagrams of *RAB5A* gene and protein and variants identified in individuals with bipolar disorder or neuropsychiatric symptoms. p.V31I, p.Q79E and p.D136N were ascertained from the BipEx database. p.Q44R was ascertained from the BaylorGenetics database of clinical exome and genome sequencing. b. Alignment of RAB5A protein variants with reference sequences. c. Bioinformatic analysis of *RAB5A* variants for pathogenicity using SIFT, PolyPhen-2 and Alpha-Missense as well as prevalence in control populations in gnomAD. SIFT scores marked in red (0.0–0.05) are predicted to be deleterious, while those marked in blue are (0.05–1.0) are predicted to be tolerated. PolyPhen-2 scores marked in red (0.909–1.0) are likely to be damaging, and scores marked in blue (0.447–0.908) are possibly damaging. Alpha missense scores marked in red (0.564–1.0) are predicted to be pathogenic.

To identify potential bipolar disorder candidate genes to screen, we interrogated BipEx (Bipolar Exomes Browser – Broad Institute)^2^ for variants in genes whose protein products physically interact with SHANK3. *RAB5A* was identified as a gene with multiple missense variants in individuals with bipolar disorder (*RAB5A*^V31I^, *RAB5A*^Q79E^, and *RAB5A*^D136N^ – identified in 2 individuals) (Fig. 1a). We further interrogated the clinical Baylor Genetics database for variants in *RAB5A* in individuals with neuropsychiatric phenotypes and identified a fourth individual with a *RAB5A* variant (*RAB5A*^Q44R^). We then aligned these variants with the reference human protein sequence as well as that of other species (Fig. 1b). *RAB5A* is not linked to human genetic disease, but there is evidence of loss-of-function constraint for the gene (gnomAD v4.1.1 pLI 0.91, o/e = 0.31, missense Z-score = 1.79)^21^. All residues with bipolar disorder-associated variants are highly conserved among vertebrates and invertebrates. The Q44 and Q79 residues lie within the switch domains which are critical for binding effector proteins^22^ while the D136 residue is within the nucleotide binding domain (NBD) which is critical for determining the identity of the bound nucleotide^23^. Finally, we used bioinformatics tools to determine the frequency of these variants in a control population (gnomAD) and evaluate the predicted impact of the variants on protein function using SIFT, PolyPhen and AlphaMissense (Fig. 1c). Based on these bioinformatic tools, all four *RAB5A* variants are predicted to be deleterious. The *RAB5A*^Q79E^ variant was identified in one individual of the non-Neuro population in gnomAD out of over 67,000 individuals while the *RAB5A*^V31I^, *RAB5A*^Q44R^ and *RAB5A*^D136N^ variants were not identified in any individuals of the control population further suggesting these variants are strong candidates for contributing to bipolar disorder.

### Impact of RAB5A bipolar disorder-associated variants in Drosophila melanogaster neuromuscular junction electrophysiology

We chose to utilize an overexpression system in *Drosophila* to investigate the functional consequences of the bipolar disorder-associated missense variants in *RAB5A*. The use of genetic tools to overexpress disease-associated variants is a common tactic in modeling human disease in *Drosophila*. Overexpression is effective in identifying functional differences between reference and disease-associated variants, and has been used to model a variety of neurological diseases, including ALS^24^, Alzheimer’s disease^25^, autism spectrum disorders^26^, and rare diseases such as *MRTFB*-associated disorder^27^. Overexpression within different tissue types can produce tractable phenotypes which can be used to better understand the mechanism of pathogenesis, even in cases where the phenotypic readout of the fly has limited similarity to the proband’s phenotype. From the bipolar disorder associated variants, we selected *RAB5A*^D136N^, *RAB5A*^Q44R^ and *RAB5A*^Q79E^ for overexpression in *Drosophila melanogaster* using the Gal4-UAS system as these three bipolar disorder-associated variants are in known functional protein domains of RAB5A (Fig. 1a). We also generated flies with the *RAB5A*^Q79L^ variant and the reference sequence of human *RAB5A* cDNA (*RAB5A*^Ref^) under the control of a UAS promoter. These constructs were used as controls. The *RAB5A*^Q79L^ variant has previously been confirmed to cause GTPase deficiency and act as a constitutively active form of RAB5A^28,29^.

**Figure 2:**
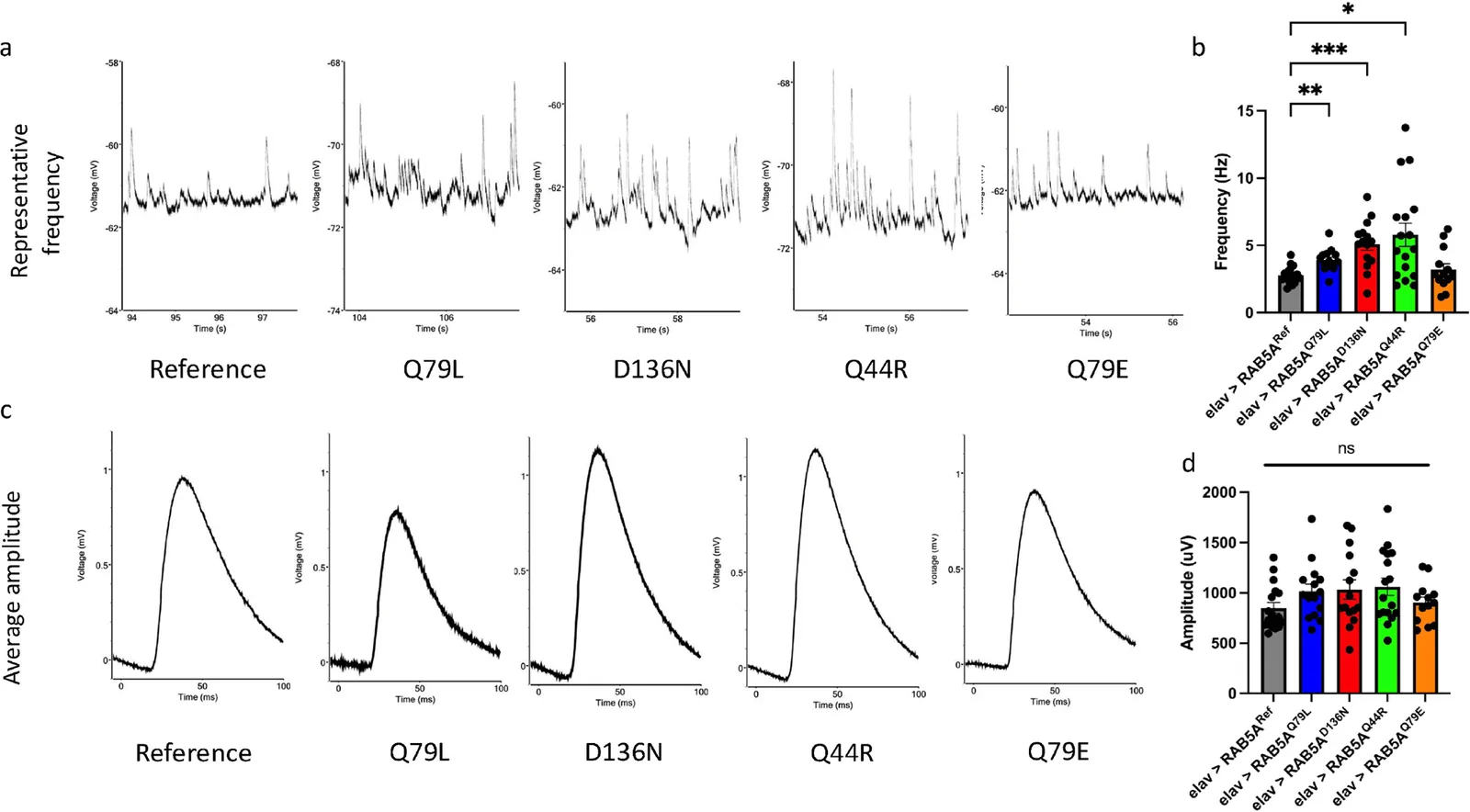
Increased synaptic neurotransmission with expression of bipolar disorder associated *RAB5A*variants in *Drosophila* neurons. a. Waveform tracings of miniature excitatory junction potentials (mEJPs) from the neuromuscular junction (NMJ) of *Drosophila* expressing reference or variant *RAB5A* driven in neurons my Elav-Gal4. b. Quantification of frequency of mEJPs from NMJs. Welch’s ANOVA test p<0.0001 with Dunnett’s multiple comparison test, *p<0.05, **p<0.01, ***p<0.001. c. Single spontaneous currents from NMJs of *Drosophila*expressing reference or variant *RAB5A* in neurons. d. Quantification of amplitude of mEJPs from NMJs. Welch’s ANOVA test, not significant.

The *Drosophila* neuromuscular junction (NMJ) is glutamatergic, has a stereotypical architecture, and is experimentally accessible, making it a model widely used to characterize synaptic function and plasticity^30^. Moreover, previous studies have shown that dominant-negative *RAB5A* mutants can impair normal endosome formation and synaptic vesicle recycling at the *Drosophila* NMJ, which can lead to a reduction in the efficiency of neurotransmitter release^11^. Therefore, variants in *RAB5A* may disrupt endosome trafficking and neurotransmitter release. To determine if *RAB5A* mutants impair neurotransmitter release, we performed electrophysiology at the *Drosophila* NMJ in flies expressing reference or variant *RAB5A* in neurons via the Elav-Gal4 driver. The frequency of miniature excitatory junction potentials (mEJPs) were significantly elevated in flies overexpressing *RAB5A*^Q44R^, *RAB5A*^D136N^ and *RAB5A*^Q79L^ variants compared with *RAB5A*^Ref^ (Fig. 2a and b). However, the frequency of mEJP did not differ with expression of the *RAB5A*^Q79E^ variant compared to the reference sequence. In contrast, there were no changes in the amplitude of mEJPs at the NMJ with any of the variants tested (Fig. 2c and d). Together, these data indicate an increase in spontaneous pre-synaptic vesicular release from neurons expressing most of the *RAB5A* bipolar disorder-associated variants.

### Impact of overexpression of RAB5A bipolar disorder-associated variants in Drosophila melanogaster endolysosomal trafficking at the neuromuscular junction

**Figure 3:**
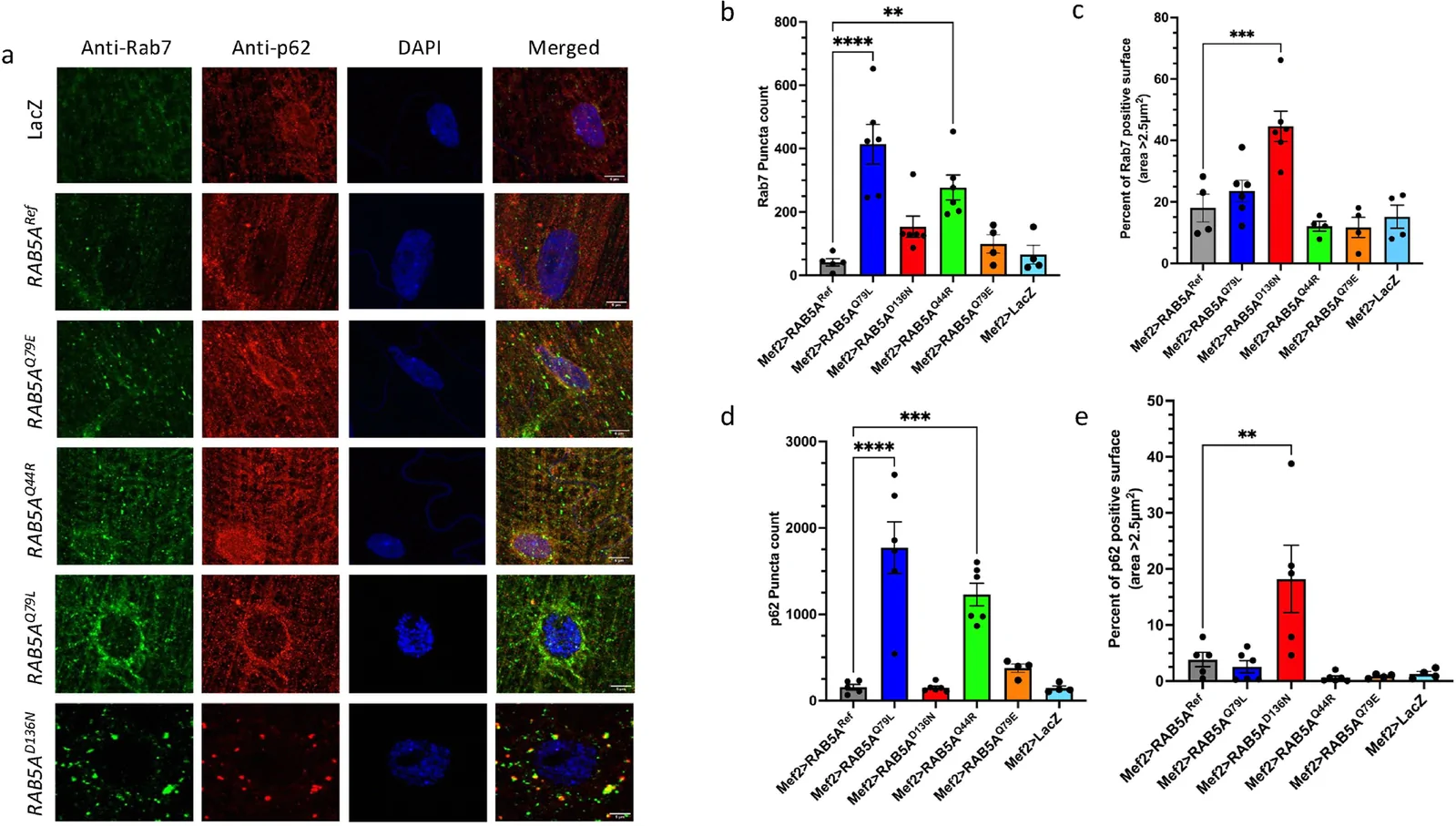
Altered endolysosomal trafficking in *Drosophila* muscle with expression of bipolar disorder associated *RAB5A*variants. a. Photomicrographs from larval body wall muscle of *Drosophila* expressing *RAB5A*reference or variant cDNA or *LacZ* cDNA by a muscle driver (Mef2-Gal4). Anti-Rab7: green, anti-p62: red, DAPI: blue. [scale bar: 5mM] b. Quantification of Rab7 puncta number from *Drosophila* larval body wall muscle of *LacZ* and *RAB5A* reference and variant cDNAs. One-way ANOVA p<0.0001, Dunnett’s multiple comparisons test **p<0.01, ****p<0.0001. c. Quantification of Rab7 puncta surface area percentage >2.5mM from *Drosophila* larval body wall muscle of *LacZ* and *RAB5A* reference and variant cDNAs. One-way ANOVA p<0.0001, Dunnett’s multiple comparisons test, ***p<0.001. d. Quantification of p62 puncta number from *Drosophila* larval body wall muscle of *LacZ* and *RAB5A* reference and variant cDNAs. One-way ANOVA p<0.0001, Dunnett’s multiple comparisons ***p<0.001, ****p<0.0001. e. Quantification of p62 puncta surface area percentage >2.5mM from *Drosophila*larval body wall muscle of *LacZ* and *RAB5A* reference and variant cDNAs. One-way ANOVA p<0.004, Dunnett’s multiple comparisons test **p<0.01.

To determine the impact of *RAB5A* bipolar disorder-associated variants on cellular processes, we evaluated the endolysosomal intracellular compartment in the *Drosophila* larval body wall. A Mef2-Gal4 driver was used to express *RAB5A* variants and reference sequence in muscle. *RAB5A* bipolar disorder-associated variants caused alterations in the number and size of late endosomes as marked by Rab7 and the autophagy adaptor protein p62 (Fig. 3). Both Rab7 and p62 puncta numbers were increased in the muscle with expression of the GTPase defective variant *RAB5A*^Q79L^ and the bipolar disorder-associated variant *RAB5A*^Q44R^ (Fig. 3a, b and d). Interestingly, the *RAB5A*^Q79L^ variant displayed perinuclear clustering of Rab7 puncta, while the *RAB5A*^D136N^ variant exhibited enlarged, perinuclear Rab7- and p62-positive puncta (Fig. 3a, c and e). This represents a striking redistribution of late endosomal and autophagic markers, with Rab7-positive vesicles clustering near the nucleus and colocalizing with p62 aggregates.

### Impact of overexpression of RAB5A bipolar disorder-associated variants in Drosophila melanogaster on viability

**Figure 4:**
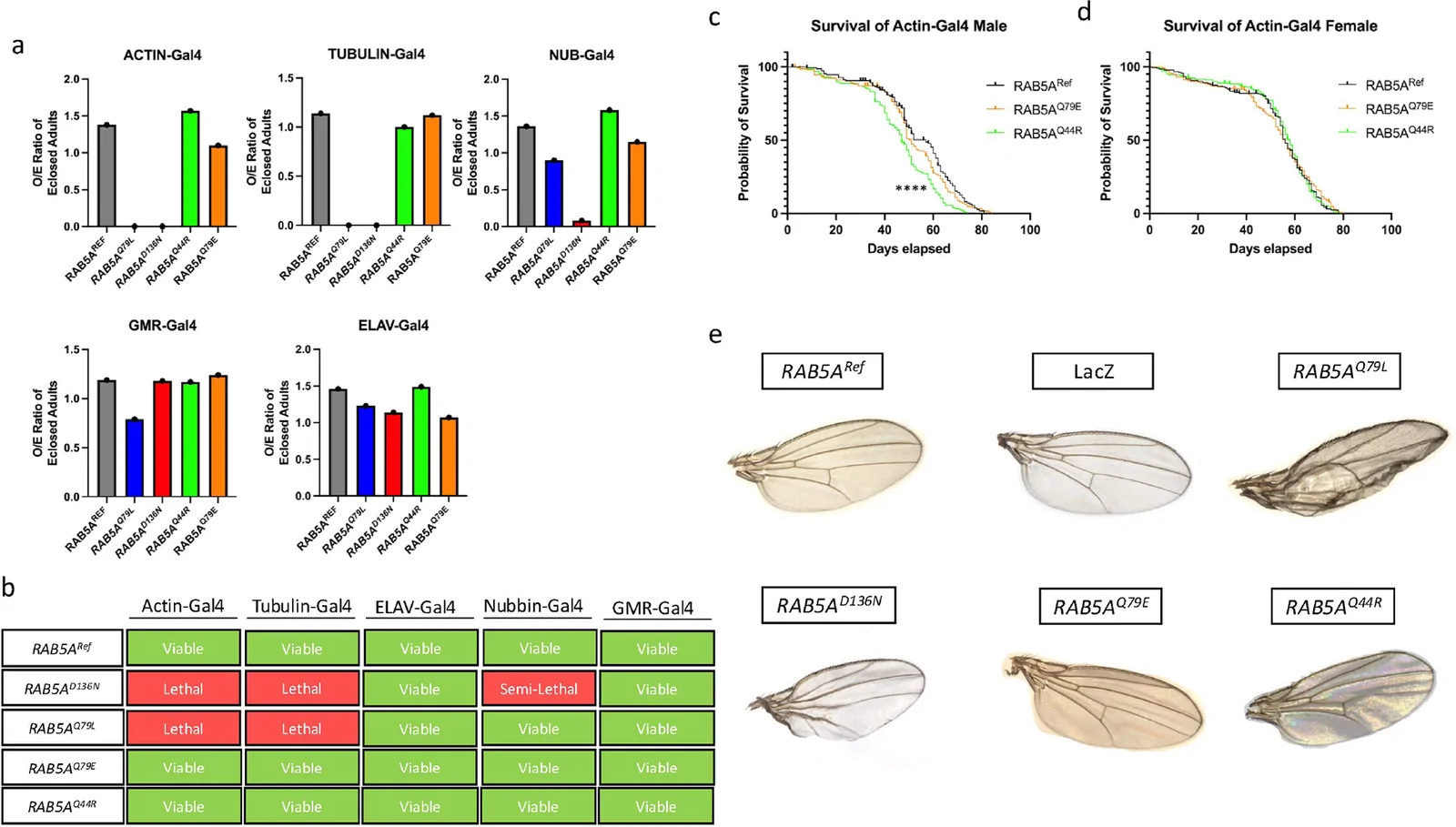
Expression of *RAB5A*bipolar disorder associated variants in *Drosophila melanogaster* impact viability, survival and wing morphology. *RAB5A* cDNA, variant or reference were driven by ubiquitous (Actin-Gal4 and Tubulin-Gal4) or tissue specific (Elav-Gal4: neuronal, Gmr-Gal4: eye, Nub-Gal4: wing) transgenes. a. Quantification of observed/expected (O/E) eclosed adults for each driver and *RAB5A*variant. b. Heat map of O/E of eclosed adults for each driver and *RAB5A*variant. Kaplain-Meyer survival plots for UAS-*RAB5A* reference, Q44R and Q79E cDNAs driven by Actin-Gal4 transgene in c. male and d. female flies. Survival analyzed with Logrank (Mantel-Cox) test with Holm-Sidak’s multiple comparison test. ****p<0.0001. e. Isolated *Drosophila* wings expressing reference or variant *RAB5A* or *LacZ* in the primordial wing driven by Nub-Gal4 transgene.

Overexpression of *RAB5A*^Ref^ by all drivers tested (Tubulin - ubiquitous, Actin - ubiquitous, Nubbin – wing, Elav – neuronal and GMR – eye) did not affect viability (Fig. 4a and b). In contrast, ubiquitous overexpression of the *RAB5A*^D136N^ and *RAB5A*^Q79L^ variants using Actin and Tubulin drivers was lethal (Fig. 4a and b). Only overexpression of the *RAB5A*^D136N^ variant in wings via a Nub-Gal4 driver led to an increase in adult lethality with preservation of the expected number of flies for all other variants using the wing driver (Fig. 4a and b). The viability of adult flies was preserved for all *RAB5A* variants when expressed in neurons and eyes via Elav-Gal4 and GMR-Gal4, respectively (Fig. 4a and b).

### RAB5A variants’ effects on lifespan in Drosophila

As several of our variants displayed significant lethality when ubiquitously overexpressed, we evaluated whether expression of nonlethal variants caused alterations in lifespan. Using the same Actin-Gal4 driver, we evaluated if ubiquitous overexpression of *RAB5*^Ref^, *RAB5*^Q44R^, *or RAB5*^Q79E^ could significantly alter the lifespan of flies. Our results demonstrated that there were sex-specific differences between male and female flies (Fig. 4c and d). For female flies, we identified no significant differences in lifespan between flies expressing *RAB5A*^Ref^ and either *RAB5A*^Q44R^ or *RAB5A*^Q79E^. In contrast, males demonstrated no significant difference between *RAB5A*^Ref^ and *RAB5A*^Q79E^ animals, but *RAB5A*^Q44R^ flies demonstrated a significant reduction in survival compared to males expressing *RAB5A*^Ref^ (*RAB5A*^Ref^ median survival 57 days versus *RAB5A*^Q44R^ median survival 47 days*)*. In aggregate, these results demonstrate that the ubiquitous overexpression of the *RAB5A*^Q44R^ variant causes a sexually dimorphic reduction in lifespan in male flies while the *RAB5A*^Q79E^ variant causes no change in lifespan for either biological sex.

### Impact of RAB5A bipolar disorder-associated variants on Drosophila wing morphology

To determine if overexpression of bipolar disorder-associated *RAB5A* variants impacted *Drosophila* morphology, all variants were expressed in the primordial wing via a Nub-Gal4 driver. Expression of the reference human *RAB5A* did not alter typical wing development (Fig. 4e). In contrast, expression of the constitutively active *RAB5A*^Q79L^ variant significantly altered wing morphology with a reduction or loss of wing veins (L2/L3/L4/L5), loss of anterior and posterior crossveins, damage to the wing margins, and blistering. Similarly, expression of *RAB5A*^D136N^ in the primordial wing impaired normal morphology with wing vein thickening, increased intervein space, brittleness, and frequent fragmentation when handled. In contrast, overexpression of *RAB5A*^Q79E^ and *RAB5A*^Q44R^ in the wing did not cause any appreciable abnormality in wing morphology.

### Impact of RAB5A bipolar disorder-associated variants on Drosophila behavior

As overexpression of *RAB5A* bipolar disorder-associated variants and reference sequence in neurons is viable, we sought to determine if their expression resulted in behavioral abnormalities. We measured climbing and bang sensitivity in flies expressing each variant and reference sequence in neurons using an Elav-Gal4 driver. Male and female *RAB5A*^D136N^ flies required a significantly longer time to recover following mechanical disruption (bang) at three days post-eclosion compared with *RAB5A*^Ref^ flies (Supplemental Fig. 1a and d). This longer recovery time was not evident in older flies (10 or 20 days post-eclosion) (Supplemental Fig. 1b, c, e, f). However, there were no consistent abnormalities in the climbing ability of flies expressing any of the *RAB5A* variants compared with reference sequence (Supplemental Fig. 2).

### Impact of overexpression of RAB5A bipolar disorder-associated variants on mammalian early endosome formation

**Figure 5:**
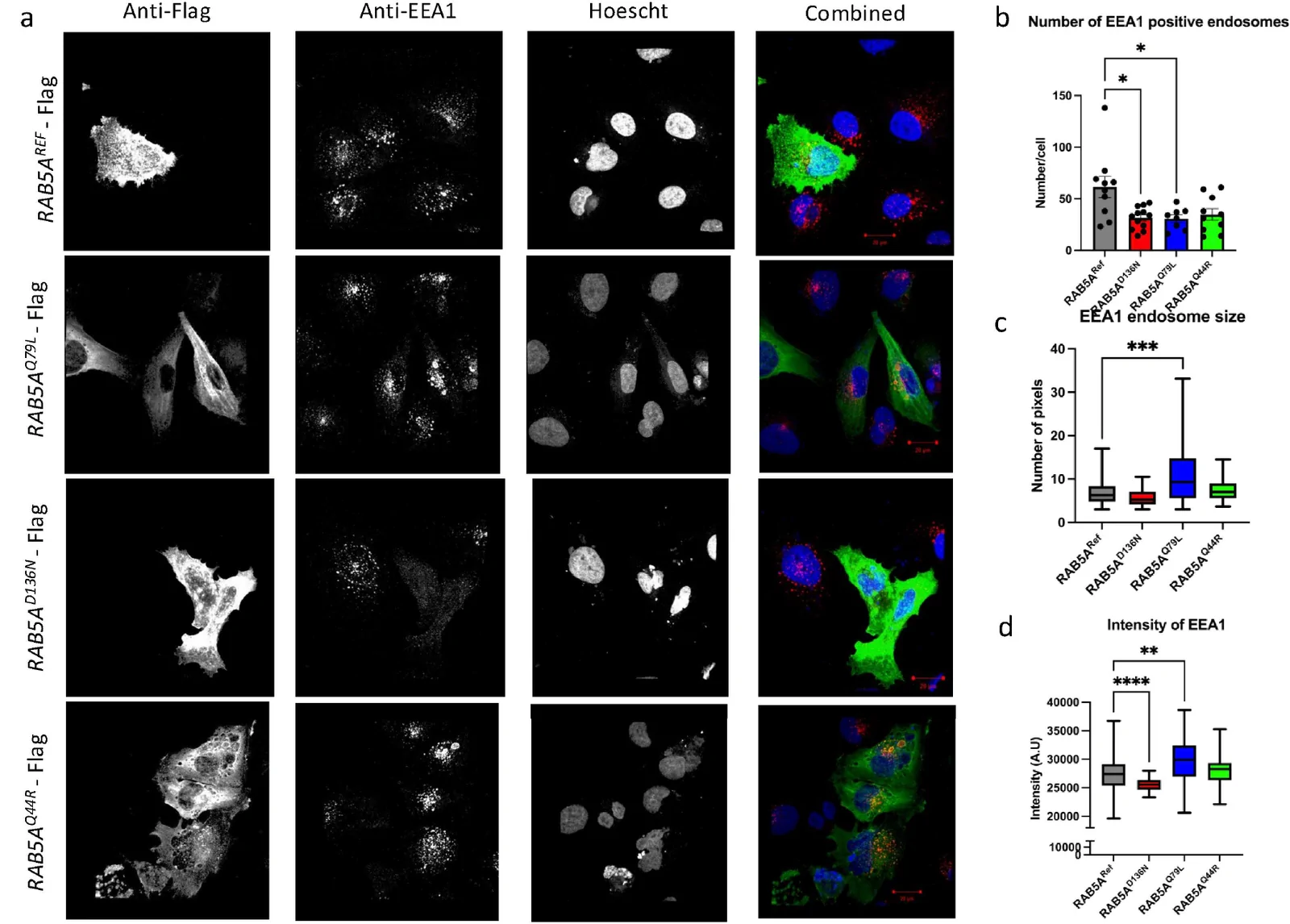
Altered early endosomal trafficking in mammalian cells with expression of bipolar disorder associated *RAB5A*variants. a. Photomicrographs of human medulloblastoma (Daoy) cells transfected with Flag-tagged expression constructs of reference or variant *RAB5A*. Anti-Flag: green, anti-EEA1: red and Hoescht 33342: blue. [scale bar: 20mm] b. Quantification of EEA1 positive endosome number in reference or variant *RAB5A* expressing Daoy cells. Kruskal-Wallis test p=0.025, Dunn’s multiple comparison test *<0.05. c. Quantification of EEA1 positive endosome size in reference or variant *RAB5A*expressing Daoy cells. Kruskal-Wallis test p<0.0001, Dunn’s multiple comparison test ***p<0.001. d. Quantification of EEA1 positive endosome intensity in reference or variant *RAB5A* expressing Daoy cells. Kruskal-Wallis test p<0.0001, Dunn’s multiple comparison test **p<0.01, ****p<0.0001.

Since multiple bipolar-associated missense variants in *RAB5A* caused extensive morphological and cellular phenotypes in *Drosophila*, we next investigated whether these same variants alter early endosomal formation in mammalian cells. Human medulloblastoma (Daoy) cells were transfected with Flag-tagged *RAB5A* harboring bipolar disorder-associated missense variants or the reference sequence. As a control, cells were also transfected with the constitutively active *RAB5A*^Q79L^ variant. Immunofluorescence was then performed to visualize those cells transfected with Flag-*RAB5A* and the early endosomal antigen EEA1. Compared with cells transfected with Flag-*RAB5A*^Ref^, cells expressing Flag-*RAB5A*^Q79L^ had fewer but larger endosomes with increased EEA1 fluorescent intensity (Fig. 5a, b, c and d). The presence of enlarged early endosomes with expression of RAB5A^Q79L^ has been previously reported^28,31^. Next, we compared early endosomes from cells expressing Flag-*RAB5A*^D136N^ with Flag-*RAB5A*^Ref^. Expression of *RAB5A*^D136N^ caused a significant reduction in early endosome number and a reduction in EEA1 intensity (Fig. 5a, b and d). Together, these data demonstrate a significant loss of early endosomes with expression of Flag-*RAB5A*^D136N^. In contrast, cells expressing Flag-*RAB5A*^Q44R^ had no significant change in number, size, or intensity of EEA1 positive early endosomes, although there was a trend toward reduction in early endosomal number (Fig. 5b).

## Discussion

There is growing evidence that endolysosomal dysfunction contributes to neurodegenerative disorders including Alzheimer’s disease and Parkinson’s disease^32,33^. Some evidence has also suggested that neuronal endolysosomal dysfunction contributes to the pathophysiology of neuropsychiatric disorders, particularly schizophrenia^34,35^. The evidence for endosomal dysfunction in schizophrenia emerges from the identification of rare variants in genes whose protein products function in endosomal trafficking^35,36^. It is hypothesized that these genomic variants lead to synaptic dysregulation; however, this remains to be fully explored.

In this study, we leveraged proteomic data to link SHANK3 (a validated bipolar disorder-associated protein) to RAB5A. Then, using large-scale genomic sequencing of individuals with bipolar disorder, we identified multiple *RAB5A* missense variants in highly conserved residues. Using a *Drosophila* overexpression model, we confirmed that these missense variants alter protein function. Specifically, the *RAB5A*^D136N^ variant causes multiple phenotypes in a tissue-dependent manner. These include abnormal wing morphology, increased frequency of vesicular release and lethality. Alterations in endosomal trafficking were also identified in both *Drosophila* NMJ preparations and cultured mammalian cells expressing *RAB5A*^D136N^.

We observed perinuclear accumulation of Rab7 and p62 with expression of *RAB5A*^Q79L^ and *RAB5A*^D136N^ in the larval body wall muscle of *Drosophila*. This is indicative of a defect in endosomal maturation and cargo turnover, as p62 build-up is a hallmark of impaired autophagic degradation^37–39^. Since Rab5 functions upstream in the early endosomal pathway and is required for the Rab5-to-Rab7 conversion step during endosome maturation^27^, its disruption likely impairs the transition to functional late endosomes/lysosomes. The resulting perinuclear clustering of Rab7 compartments, together with persistent p62 accumulation, suggests that although late compartments are generated, their trafficking or degradative capacity is compromised, leading to defective autophagic flux. We also determined that the *RAB5A*^D136N^ variant also disrupts early endosome compartments positive for EEA1 in a mammalian cell system. Together, these data reveal that bipolar disorder-associated variants, and in particular the *RAB5A*^D136N^ variant, disrupt normal endolysosomal trafficking.

The function of RAB5A in neuronal activity has only begun to be elucidated. RAB5A has been identified in both the axonal and somatodendritic compartments of neurons^40^. Moreover, RAB5A is known to be critical for maintaining neuronal polarity by proper sorting of excitatory neurotransmitter receptors to the somatodendritic compartment^41^. Overexpression of a dominant negative version of RAB5A was found to block the proper sorting of excitatory neurotransmitter receptors. Finally, RAB5A was discovered to be critical for physiologic internalization of D2 dopamine receptors in COS7 cells^29^. Together, these data support a critical role for RAB5A in endocytic trafficking of neurotransmitter and neuromodulatory receptors to the synaptic surface. Future work will be critical to determine how bipolar disorder-associated variants in RAB5A alter trafficking of these receptors.

The impact of the *RAB5A*^D136N^ variant on biochemical function of the resultant RAB5 protein has been previously investigated^23^. The p.D136N amino acid substitution led to a preference for xanthine nucleotides over guanine. This work was undertaken solely based upon theoretical changes in nucleotide binding of RAB5 and did not seek to determine the biological impact of the variant *ex vivo* in cells in culture or *in vivo* in living organisms. Although the current work clearly demonstrates bipolar disorder-associated variants in *RAB5A*, most severe with *RAB5A*^D136N^, impair normal endolysosomal function, the precise mechanism whereby these variants lead to *in vivo* phenotypes such as increased synaptic neurotransmission requires further investigation. Our data relied on overexpression models both in *Drosophila* and mammalian cells. Future studies of the impact of these variants will require their introduction into the germline of experimental systems to overcome this limitation.

Expressing human cDNAs harboring variants identified in individuals with neurodevelopmental disorders has proven fruitful for confirming the pathogenicity of novel variants and genes for disorders with early developmental phenotypes. To our knowledge, this approach has not been used to validate variants identified from individuals with neuropsychiatric disorders such as schizophrenia and bipolar disorder. From this work on *RAB5A* variants identified in individuals with bipolar disorder, we propose this could be a useful approach for relatively high throughput screening of functional consequences of such genomic variants.

## METHODS

### Identification of RAB5A variants and bioinformatic analyses

*RAB5A* variants were identified from the Bipolar Exome Database hosted at the Broad Institute (https://bipex.broadinstitute.org) or from the clinical exome and genome database from Baylor Genetics. Alignment of *RAB5A* variants with human and other organism reference sequences was performed using SnapGene. Variant bioinformatic characterization for pathogenicity was performed using online tools: SIFT (https://sift.bii.a-star.edu.sg/www/code.html), PolyPhen-2 (http://genetics.bwh.harvard.edu/pph2/) and AlphaMissense (https://alphamissense.hegelab.org).

### Drosophila Methods

#### Housing and handling

All fly strains used in this study were generated in the Wangler lab or obtained from the Bloomington *Drosophila* Stock Center (BDSC). All flies were grown in a temperature and humidity-controlled incubator. All animals were maintained at 50% humidity on a 12-hour light/dark cycle. Flies were allowed to feed *ad libetum* on standard fly food medium consisting of water, yeast, soy flour, cornmeal, agar, corn syrup, and propionic acid. Collection of flies for experiments was performed during the fly’s subjective daylight hours.

#### Generation of UAS-human cDNA lines

UAS lines were generated as previously described in prior publications^26,42,43^ Briefly, reference human cDNA clones corresponding to the NM_004162.4 transcript were obtained in a pDONR221 vector through the Kenneth Scott cDNA Collection at Baylor College of Medicine. Variants were generated in the pDONR vector via site directed mutagenesis with the Q5 mutagenesis kit (NEB) and verified by sequencing. Mutagenesis primers were derived from NEBaseChanger. LR clonase II (ThermoFisher) enzyme was used to transfer sequence verified reference and variant ORF’s into the p.UASg-HA.attB destination vector. Verified ORF’s in the p.UASg-HA.attB vector were microinjected into ~ 200 embryos and transferred into an attP docking site (VK00037) via ΦC31 mediated transgenesis.

### Drosophila neuromuscular junction electrophysiology

Wandering 3rd instar *Drosophila* larvae were removed from the side of the vial, immersed in Ca^2+^ free HL3 solution (70mM NaCl, 5mM KCl, 20mM MgCl_2_, 10mM NaHCO_3_, 5mM trehalose, 115 mM sucralose, and 5 mM HEPES), and pinned into the bottom of a petri dish containing several millimeters of sylgard. A single vertical incision was made between the two tracheal tubes and paired cuts were made to allow the larval muscle wall to lay flat against the sylgard at the bottom of the dish. All internal organs were removed with forceps. Intracellular recordings were performed in HL3 solution containing 1mM Ca^2+^ at 20–22°C. Microelectrodes were pulled from borosilicate glass with a micropipette puller (Stutter Instruments P-97) and filled with 3M KCl. Only micropipettes with a resistance between 10–20 MΩ were used. Spontaneous miniature excitatory junction potentials (mEJPs) were recorded from muscle 6 segment A3 and A4 with an Axoclamp 900A amplifier (Molecular Devices) set to bridge mode and a Digidata 1550 digitizer (Molecular Devices). Recordings were collected with pClamp10.4 software (Molecular Devices) and resulting traces were analyzed with AxoGraph X software. Miniature EJPs were recorded over a period of 120 seconds. Only cells with a resting potential between − 50 and − 70 mV and an input resistance of > 4mΩ were used for analysis.

### Immunocytochemistry in Drosophila

The 3rd instar wandering larvae were dissected in 1X PBST (1X phosphate-buffered saline [Gibco, 10010–023] with 0.5% Tween20 [Fisher Scientific, BP337–500]) on sylgard plates [sylgard 184, Electron microscopy sciences]. Dissected larvae were fixed with 4% paraformaldehyde for 10 min at room temperature (RT) followed by three washes with 1X PBST for 10 min each. Larval samples blocking was performed in 5% normal goat serum (NGS) prepared in 1X PBST for 30 minutes at room temperature to reduce non-specific antibody binding. Samples were incubated overnight at 4°C with primary antibodies followed by three washes with 1X PBST for 10 min each. Samples were then incubated overnight at 4°C with the secondary antibody followed by three washes with 1X PBST. Samples were then mounted on slides using Vectashield containing DAPI (Vector laboratories, H-1200– 10). The following primary antibodies were used: rabbit anti-SQSTM1/p62 (1:500) and mouse anti-Rab7 (0.5 μg/ml, Developmental Studies Hybridoma Databank, Rab7s). The primary rabbit anti-SQSTM1/p62 anti-peptide antibodies (used at 1:100 dilution) was generous gift from McNew lab, Rice University. The following secondary antibodies were used: Donkey anti-rabbit IgG conjugated to Cy3 (Jackson ImmunoResearch, Cat. #711–165-152, 1:1000) and Donkey anti-mouse IgG conjugated to Alexa fluor 647 (Jackson ImmunoResearch, Cat. # 715–606-150, 1:1000).

#### Imaging larval body wall muscle

High-resolution imaging was performed using a Zeiss LSM800 Airyscan confocal microscope (Carl Zeiss Microscopy GmbH, Germany) equipped with Plan-Apochromat 63×/1.2 NA water immersion objective. Images were acquired with a frame size of 1024 pixels to ensure optimal resolution. Fluorophores were excited and detected using the specific laser lines and spectral ranges.

Raw image data were processed using the Airyscan Processing module in ZEN 2.6 software (Blue edition). The 2D Super-Resolution (SR) mode was applied to enhance spatial resolution beyond the diffraction limit. Deconvolution was carried out using the built-in Wiener filter, with the filtering parameter set to 'Standard' to balance signal restoration and noise suppression. All processing settings were maintained constantly across all experimental conditions to ensure consistency and comparability of results.

### Image quantification of Drosophila Rab7 and p62

Quantitative analysis of fluorescence images for tissue sections stained with anti-p62 and anti-Rab7 were performed using the Surface module of the Imaris software (version 9.8.2; Bitplane, Zurich, Switzerland). In this module, 3D surfaces were rendered for the specific fluorophore signals based on intensity, using the background subtraction option to enhance signal specificity. To isolate individual puncta an area-based filtering strategy was applied. A lower threshold limit of 0.1 μm^2^ for p62 and 1 μm^2^ for Rab7 were set, whereby only surfaces with an area equal to or greater than 0.1 μm^2^ for p62 and area equal to or greater than 1 μm^2^ for Rab7 were retained for further analysis. This optimized thresholding approach was essential to minimize background noise and reduce the incidence of false-positive surface detection. The data was analyzed for puncta number and size distribution for both p62 and Rab7.

#### Imaging of wing morphology

*Drosophila* wings were removed under CO_2_ anesthesia and placed onto slides for imaging. Images were obtained using a Leica MZ16 stereomicroscope equipped with an Optronics MicroFire Camera and Image Pro Plus 7.0 software to perform extended depth of field images.

##### Bang sensitivity and climbing assessment

Flies intended to be used for trials were identified on the day of eclosure and group housed with other flies of the same genotype. Assessments were made at 0–3 days, 10 days, and 20 days post eclosure from the pupal case. Climbing assays were performed by tapping the flies to the bottom of the vial 3 times by hand, then observing the flies as they climbed towards a marker on the vial 8cm above the vials base. The time required to reach the marker was recorded using a digital stopwatch. Any flies which failed to reach the marker within 60 seconds were recorded as requiring 60 seconds, and the trial stopped. For bang sensitivity, flies were vortexed in a standard fly vial (lacking food). Flies were vortexed at full speed (Fisher STD Vortex Mixer, Cat. No: 02215365) for 10 seconds. Recovery times were recorded using a digital stopwatch. Both bang and climbing assays were performed on a minimum of 20 flies.

##### Assessment of lethality, survival and morphological phenotypes

Crosses for the assessment of lethality, survival and morphology were performed using -Gal4 drivers as indicated in the text. 5–10 virgin females were crossed to a similar number of males. Parents were transferred to a new vial after 5–7 days to collect multiple generations of progeny and to prevent accidental selection of parental flies for assessment. After 3 passages, any surviving parents were discarded. To assess lethality, flies were collected daily, and the total number of adult flies was scored based on the presence or absence of balancers. Any morphological changes were noted in surviving animals lacking balancers, and flies with morphological changes were isolated for imaging if the phenotype appeared in more than 70% of the progenies. For lethality assessment, a minimum of 70 flies were scored. Viability was calculated via an evaluation of the number of observed progenies compared to the number of expected progenies based on mendelian ratios (O/E ratios). Animals were classified as lethal if the O/E ratio was less than 0.15, and semi-lethal if the O/E ratio was less than 0.8. For lifespan assays, at least 100 flies per genotype were collected after eclosion in vials containing 10 flies each. The number of surviving animals was measured every other day, and the vial was changed to avoid loss of experimental animals to getting stuck in the food substrate. The survival rates of the genotypes were subsequently compared after the death of the last fly.

#### Mammalian Cell Culture

Human medulloblastoma (Daoy) cells were cultured in Dulbecco’s Modified Eagle Media (DMEM) supplemented with 10% heat inactivated fetal bovine serum in a 37°C incubator supplemented with 5% CO_2_. Flag-*RAB5A* was a gift from Qing Zhong (Addgene plasmid #28043; http://n2t.net/addgene:28043 ; RRID:Addgene_28043). Missense variants were introduced using the Q5 Site-Directed Mutagenesis Kit (New England Biolabs). For transfection of Flag-*RAB5A* constructs, 150,000 Daoy cells were plated per chamber of a 4-chamber cell culture slide (Millipore). Transfection was performed using the Lipofectamine 3000 reagent (Thermo Fisher),according to manufacturer’s protocol. For each transfection, 5μg of DNA was combined with 10μL of P3000 in 125μL of Optimem. In a separate tube, 20μL of Lipofectamine 3000 was added to 650μL of Optimem. The DNA and 125μL of the Lipofectamine solutions were then combined and incubated for ten minutes at room temperature. The liposomal particles were then added to Daoy cells in the 4-chamber culture slide and incubated for 48 hours at 37°C/5%CO_2_. After 48 hours, the slides were fixed and stained.

#### Immunofluorescent staining of cultured mammalian cells

Daoy cells were washed in Phosphate Buffered Saline (PBS) then fixed in 4% sucrose buffered formaldehyde for twenty minutes at room temperature. Cells were then washed again in PBS twice for 5 minutes and then permeabilized in PBS-T (0.1% Triton X-100) for 10 minutes at room temperature. Cells were then blocked in 5% donkey serum in PBS for 30 minutes at room temperature. Primary antibody hybridization was then performed overnight at 4°C with rocking with anti-Flag (Sigma-Aldrich F1804) and EEA1 (Fisher Scientific PIMA514794) diluted 1:200 in 5% donkey serum in PBS. After primary antibody hybridization, cells were washed in PBS for 5 minutes with rocking three times. Secondary hybridization was performed at room temperature for approximately two hours with secondary antibodies diluted 1:200 in 5% donkey serum in PBS. Anti-Flag was detected with anti-mouse Alexa 488 (Invitrogen) and anti-EEA1 was detected with anti-rabbit Alexa 546 (Invitrogen). After secondary hybridization, cells were washed twice with PBS for 5 minutes each then with Hoescht 33342 (Thermo Scientific) diluted 1:1000 in PBS for 10 minutes at room temperature and with a final PBS wash. A coverslip was mounted in Prolong Diamond (Invitrogen) and the coverslip sealed after 24 hours.

#### Imaging and image analysis in mammalian cells

Transfected Daoy cells were imaged on a Zeiss 880 confocal microscope. Imaging parameters were set with Daoy cells transfected with reference Flag-*RAB5A* and unchanged for imaging of cells transfected with each of the variants. Images were analyzed in Image J. For quantification of number, intensity and size of EEA1 positive endosomes, signal threshold and minimum particle size were the same for cells transfected with all Flag-*RAB5*A constructs.

## Supplementary Material

Supplementary Files

This is a list of supplementary files associated with this preprint. Click to download.

• supplementalfigure2.pdf

• supplementalfigure1.pdf
